# Human immune globulin 10% with recombinant human hyaluronidase in multifocal motor neuropathy

**DOI:** 10.1007/s00415-019-09475-x

**Published:** 2019-07-19

**Authors:** Ingrid J. T. Herraets, Jaap N. E. Bakers, Ruben P. A. van Eijk, H. Stephan Goedee, W. Ludo van der Pol, Leonard H. van den Berg

**Affiliations:** 1 Department of Neurology and Neurosurgery, UMC Utrecht Brain Center Rudolf Magnus, Heidelberglaan 100, 3584 CX Utrecht, The Netherlands; 2 Department of Rehabilitation, Brain Center Rudolf Magnus, UMC Utrecht, Heidelberglaan 100, 3584 CX Utrecht, The Netherlands; 3 Biostatistics and Research Support, Julius Center for Health Sciences and Primary Care, University Medical Center Utrecht, Heidelberglaan 100, 3584 CX Utrecht, The Netherlands

**Keywords:** Peripheral neuropathy, Multifocal motor neuropathy, Immunoglobulin, Treatment effect, Safety

## Abstract

**Objective:**

The primary aim was to determine the safety of treatment with human immune globulin 10% with recombinant human hyaluronidase (fSCIg) compared to intravenous immunoglobulin (IVIg) in a prospective open-label study in patients with multifocal motor neuropathy (MMN).

**Methods:**

Our study consisted of two phases: the IVIg phase (visits 1–3; 12 weeks), in which patients remained on IVIg treatment, and the fSCIg phase (visits 4–7; 36 weeks), in which patients received fSCIg treatment. After visit 3, IVIg was switched to an equivalent dose and frequency of fSCIg. Outcome measures were safety, muscle strength, disability and treatment satisfaction.

**Results:**

Eighteen patients were enrolled in this study. Switching to fSCIg reduced the number of systemic adverse events (IVIg 11.6 vs. fSCIg 5.0 adverse events/per person-year, *p* < 0.02), and increased the number of local reactions at the injection site (IVIg 0 vs*.* fSCIg 3.3 local reactions/per person-year, *p* < 0.01). Overall, no significant differences in muscle strength and disability between fSCIg and IVIg were found. Treatment with fSCIg was perceived as optimal treatment option by 8 of the 17 patients (47.1%) and they continued with fSCIg after study closure because of improved independence and flexibility to administer treatment.

**Conclusion:**

Treatment with fSCIg can be considered a safe alternative for patients with MMN on IVIg treatment. fSCIg could be a favorable option in patients who prefer self-treatment and more independency, and in patients who experience systemic adverse events on IVIg or have difficult intravenous access.

**Electronic supplementary material:**

The online version of this article (10.1007/s00415-019-09475-x) contains supplementary material, which is available to authorized users.

## Introduction

Multifocal motor neuropathy (MMN) is an immune-mediated demyelinating neuropathy characterized by asymmetric muscle weakness, predominantly of the upper limbs [1–3]. Men are more commonly affected as woman with a ratio of 2.6:1 [1, 2]. In most patients, the first symptoms occur between age 20 and 50 years [1]. Various trials have shown a beneficial effect of intravenous immunoglobulins (IVIg) on muscle strength in MMN and a comparable effect of subcutaneous immunoglobulins (SCIg) [4–7].

Although a large number of studies have demonstrated that IVIg treatment is well tolerated, various systemic adverse events have been reported: the majority, such as headache, malaise and chills, are transient and relatively mild, but some rare adverse events, such as anaphylactic and skin reactions, are serious [4]. Moreover, repeated venous access and administration in hospital or at home, in the presence of a nurse, is a burden for the patient. SCIg treatment is considered a good alternative as it can be administered by the patient or informal caregiver and produces fewer systemic adverse reactions [5, 8]. However, limitations of subcutaneous infusion volumes and reduced bioavailability require more frequent infusion and an increase in dose in approximately 50% of the patients [5].

A relatively new treatment that overcomes the disadvantages of the conventional SCIg is human immune globulin 10% with recombinant human hyaluronidase (fSCIg). Subcutaneous administration of hyaluronidase increases SCIg dispersion and absorption and, therefore, provides higher doses of SCIg with less frequent infusion and with the benefit of a higher bio-availability [9–11]. Treatment with fSCIg has been approved by the Food and Drug Administration (FDA) for primary immunodeficiency (PID), but not for inflammatory neuropathies including MMN. This study explores the safety and treatment satisfaction of fSCIg compared to IVIg in patients with MMN.

## Methods

### Study design and patients

This prospective, open-label study was performed between November 2016 and February 2018 in the UMC Utrecht, a tertiary referral center for neuromuscular disorders. Patients with the diagnosis of MMN according to the EFNS/PNS criteria, who had been stable on IVIg therapy for ≥ 1 year, were eligible for inclusion in this study. Exclusion criteria for this study were: (1) treatment with other immunosuppressive drugs (e.g., cyclophosphamide, azathioprine, cyclosporine) in the 6 months preceding the study, (2) age < 18 years, and (3) female patient pregnant or breast-feeding. The study protocol was approved by the local medical ethics committee Utrecht (METC Utrecht; file ID NL52642.041.15) and registered in the Eudra-CT (2015-000828-28) and clinicaltrial.gov (NCT02885259) databases and has, therefore, been performed in accordance with the ethical standards laid down in the 1964 Declaration of Helsinki and its later amendments. All patients gave written informed consent.

### Outcome measures

**Fig. 1:**
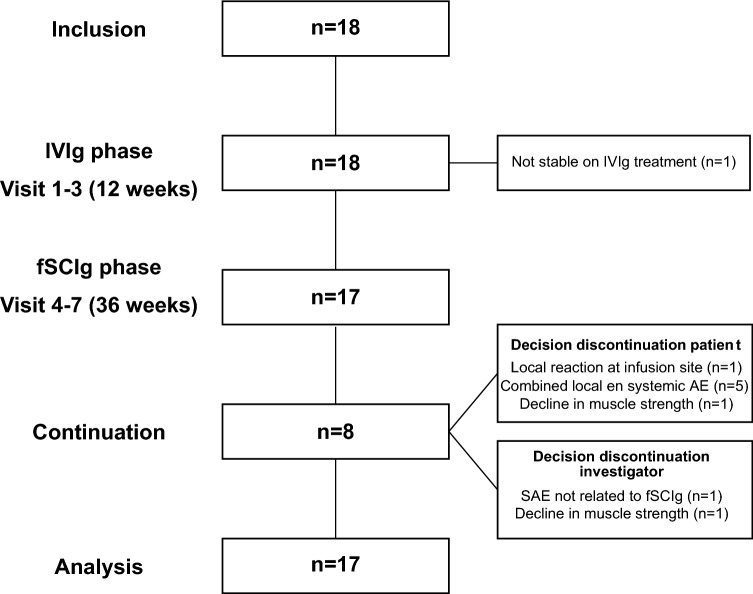
Flowchart study. *fSCIg* human immune globulin 10% with recombinant human hyaluronidase, *IVIg* intravenous immunoglobulins, *SAE* serious adverse event, *AE* adverse event

**Fig. 2:**
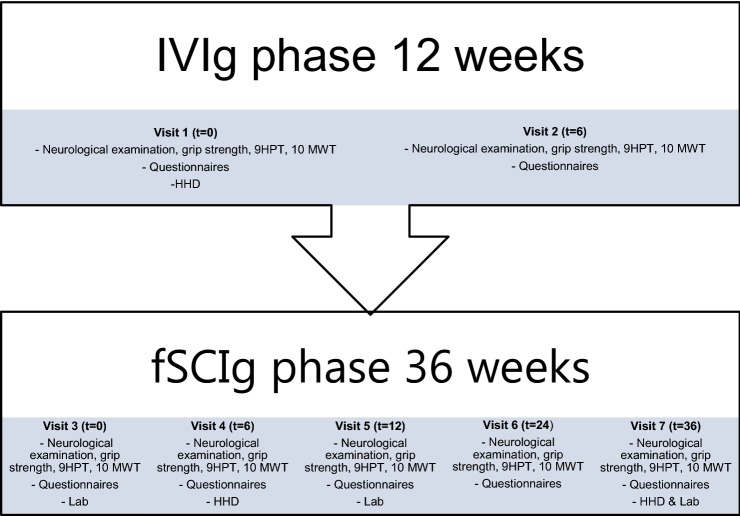
Outcome measures collected per visit. Questionnaires consisted of a standardized questionnaire for adverse events, treatment satisfaction rated on a 0–10 VAS scale, Guy’s Neurological Disability Scale and Self-Evaluation Scale. *fSCIg* human immune globulin 10% with recombinant human hyaluronidase, *HHD* hand-held dynamometry, *IVIg* intravenous immunoglobulins, *Lab* laboratory tests, *t* time in weeks, *9 HPT *9-Hole Peg test, *10 MWT* 10-meter walk test

This study consisted of two successive phases: the IVIg phase lasting 12 weeks and the fSCIg phase of 36 weeks (Fig. 1). During the IVIg phase, patients visited the outpatient clinic every 6 weeks (visit 1–3). In the fSCIg phase, patients visited the outpatient clinic on weeks 18 (visit 4), 24 (visit 5), 36 (visit 6) and 48 (visit 7). At each visit, all outcome measures were collected, except for hand-held dynamometry (HHD) (visits 1–4–7) and laboratory tests (visits 3–5–7) (Fig. 2).

The primary aim was to assess the safety of fSCIg treatment. During the study, we documented safety using a standardized questionnaire that included a number of adverse events and laboratory tests, including hemoglobin, hematocrit, haptoglobin, reticulocytes, lactate dehydrogenase, bilirubin, and direct Coombs test to exclude hemolytic anemia due to fSCIg. In addition, blood samples were obtained to explore a possible association between rHuPH20-binding antibody positivity and adverse events. In case of a serious adverse event related to fSCIg, the study treatment had to be discontinued. If a patient experienced an adverse event, the investigator or the patient him/herself could decide to discontinue the study treatment and resume regular IVIg treatment.

The second aim of this study was to measure muscle strength. All patients underwent a standardized neurological examination, and motor function of 18 muscle groups (abduction of the arm, flexion and extension of the forearm, wrist and fingers, spreading of the fingers, abduction, adduction and opposition of the thumb, flexion of the hip, flexion and extension of the knee, and flexion and extension of the foot and toes) was graded bilaterally using the Medical Research Council (MRC) scale to calculate the MRC-sum score. Grip strength was determined bilaterally with the Martin-Balloon-Vigorimeter (Firma Gebrüder Martin, Tuttlingen, Germany) and measured in Kilopascals (kPa). Hand-held dynamometry (HHD) was performed bilaterally in nine muscle groups (abduction of the arm, flexion of the forearm, extension of the wrist and fingers, spreading of the fingers, abduction of the thumb, flexion of the hip, and extension of the foot and big toes) by a physiotherapist using the microFET2 (Hoggan health industries, Draper, UT, USA). Muscle strength with HHD was measured in Newton (N).

In addition, disability was determined with the Guy’s Neurological Disability Scale and Self-Evaluation Scale (SES). To measure hand function and finger dexterity, the 9-Hole Peg Test (9-HPT) was performed with the most affected hand and the mean duration (in seconds) of five subsequent trials was calculated. Walking was evaluated with the 10-meter walk test (10 MWT), for which the mean duration (in seconds) and number of steps of three repeats was calculated. Finally, patients were asked to rate their treatment satisfaction on a 0–10 point VAS-scale.

### Treatment protocol

During the IVIg phase, patients remained on their regular IVIg maintenance therapy regimen to determine their current neurological functioning on therapy. After completion of the IVIg phase, patients switched to fSCIg treatment at a dose and frequency equivalent to the IVIg dose and frequency. Both human immune globulin 10% and recombinant human hyaluronidase were infused using a Micrel Rythmic pump. Personalized titration schedules were devised to increase the dose of fSCIg slowly and thus allow patients to get used to the presence of fluid in their abdominal wall. In general, patients received a dose of 25% of fSCIg in week 1, of 50% in week 2 and their total dose of fSCIg in week 3. IVIg treatment was discontinued when the total dose of fSCIg was administered. Treatment with fSCIg was administered in the patients’ home setting. Specialized nurses were present during the first six infusions to teach patients how to administer fSCIg and to monitor and treat potential adverse events. After the first six infusions, patients were allowed to self-administer fSCIg at home.

If patients developed a decline in muscle strength during fSCIg treatment, investigators could increase the dose of fSCIg, provided there was no increase in adverse events. This decline in muscle strength was defined as a worsening of ≥ 1 of the outcome measures: Guy’s Neurological Disability Scale (increase ≥ 1 in either the upper or lower limb score), SES (an increase of ≥ 1 at ≥ 2 motor activities) and HHD (a decrease of 50% in ≥ 2 clinically affected muscles groups). If patients showed no improvement after increasing fSCIg dose, or if adverse events occurred, fSCIg maintenance treatment was discontinued and IVIg treatment resumed.

### Statistical analysis

All data were summarized using the median and range for continuous variables and number and percentage for categorical variables. Clinical characteristics between patients that continued with fSCIg or discontinued were compared using the Fisher’s exact test for categorical variables and the Mann–Whitney *U* test for continuous variables. The absolute frequency of adverse events with IVIg and fSCIg were compared using Fisher’s exact test. For each patient, we determined whether he or she switched back to IVIg, and, if so, the time spent on fSCIg. This time-to-event variable was visualized using Kaplan–Meier curves. Subsequently, we assessed which baseline factors affected the time spent on fSCIg using a Cox proportional hazards model. The mean difference of the HHD measurement was calculated as the difference between first evaluation under fSCIg (visit 4) and baseline (visit 1) and analyzed using a paired *t* test. The longitudinal outcome measures were analyzed using linear mixed effect models (LMMs). The dependency in the data due to the repeated measures was accounted for by a random intercept per individual. The fixed effects part contained a term for treatment (IVIg or fSCIg) and a term for time (in months). Significance of both factors was determined using the likelihood ratio test. Due to the exploratory nature of this study, we did not adjust for multiple testing and results were considered significant when the *p* value was lower than 0.05. All analysis were conducted in SPSS 22 (SPSS Inc., Chicago IL, USA) except for the LMMs that were fitted using the lmer function in the R package lme4 (version 1.1–12) [12].

## Results

### Patients

The MMN database of the UMC Utrecht was screened (*n* = 130) and all patients fulfilling the inclusion criteria were invited for participation (*n* = 102). Of these, 54 patients did not respond or could not be reached, and 30 patients declined participation. In total, 18 patients, all treated with IVIg in home setting, were enrolled in this study between November 2016 and May 2017. Clinical characteristics of participants (*n* = 18) and non-participants (*n* = 30) were not significantly different, except for disease duration (6.7 years vs. 16.9 years). One patient appeared to be unstable on IVIg treatment during the IVIg phase and was excluded from the study. The baseline characteristics of the remaining 17 patients are provided in Table 1. Two patients were lost to follow-up, both at visit 4 after discontinuation of fSCIg. In one patient, visit 4 was missing because of surgery for a hernia. According to the protocol, an increase of dose was required in one patient on IVIg treatment and in three patients on fSCIg treatment.

**Table 1:** Baseline characteristics

	Total cohort (*n* = 17)	Continuation fSCIg (*n* = 8)	Discontinuation fSCIg (*n* = 9)	*p * value
Age at inclusion (years)	57.7 (36.5–69.5)	61.6 (36.5–69.5)	50.2 (46.2–68.9)	0.16
Sex (male)	14 (82.4)	7 (87.5)	7 (77.8)	> 0.99
Symptom duration (years)	6.9 (2.0–29.9)	6.6 (2.0–29.9)	10.2 (4.9–23.9)	0.67
Duration of IVIg therapy (years)	4.9 (1.2–23.8)	4.3 (1.2–23.8)	4.9 (1.2–13.5)	0.88
Dosage IVIg (g/kg)	0.5 (0.3–2.2)	0.4 (0.3–2.2)	0.6 (0.4–0.6)	0.37
Interval IVIg (days)	21 (7.0–35.0)	21.0 (7.0–28.0)	21.0 (7.0–35.0)	0.37
Abnormal CSF protein	5/6 (83.3)	3/4 (75.0)	2/2 (100.0)	> 0.99
Abnormal MRI brachial plexus	6/10 (60.0)	2/5 (40.0)	4/5 (80.0)	0.52
Presence of anti-GM1 autoantibodies	11/16 (68.8)	7/8 (87.5)	4/8 (50.0)	0.28

Data are shown for the total cohort (*n* = 17) and for patients that continued with fSCIg (*n* = 8) and discontinued with fSCIg (*n* = 9). Data are in median (range) or *n* (%)

CSF, cerebrospinal fluid; fSCIg, human immune globulin 10% with recombinant human hyaluronidase; g/kg, grams per kilogram; IVIg, intravenous immunoglobulins

### Reasons and determinants of discontinuation

Nine patients (52.9%) discontinued fSCIg during the treatment phase after an average number of infusions of 4.7 (SD: 4.6). Baseline characteristics of patients that continued with fSCIg (*n* = 8) and discontinued (*n* = 9) were not significantly different (Table 1). Six participants decided to discontinue because of adverse events [local reactions at the injection site (*n* = 6), nausea (*n* = 1), cramps (*n* = 1), general malaise (*n* = 2) and headache (*n* = 1)]. One patient showed a decline in muscle strength but refused to increase the dose of fSCIg and chose to switch back to IVIg. The investigators withdrew two participants because of an unrelated serious adverse event (ischemic stroke, *n* = 1) and decline in muscle strength despite increasing the dose of the fSCIg (*n* = 1) (Fig. 1).

We evaluated which outcome measures were associated with treatment discontinuation (i.e. treatment satisfaction, Guy’s Neurological Disability score, SES, 10 MWT and 9-HPT). Interestingly, treatment satisfaction was the only baseline factor associated with continuation of fSCIg: a higher satisfaction during the IVIg phase of the trial was associated with the continuation of fSCIg (HR 0.31, 95% CI 0.12–0.83, *p* = 0.007). To exemplify: after 6 months, 78% of the patients, whose satisfaction with IVIg treatment was initially ≥ 8, remained on fSCIg, compared to 25% of patients with a satisfaction rate < 8 (Online Resource 1).

### Safety

Frequencies of adverse events, adverse events per year and adverse events per patient are shown for IVIg and fSCIg in Table 2. The frequency of systemic adverse events was lower in fSCIg (*n* = 87 on IVIg vs*.* n = 35 on fSCIg, *p* = 0.04); headache and general malaise occurred less often in fSCIg (*p* < 0.01; *p* < 0.01), while cramps and local reactions at the injection site occurred more often (*p* = 0.03; *p* < 0.01). Neither of the patients developed hemolytic anemia, nor did any develop rHuPH20-binding antibodies after initiation of fSCIg treatment.

**Table 2:** Safety profile of IVIg and fSCIg

	IVIg		fSCIg		*p * value
	Frequency	Rate	Frequency	Rate	
Any systemic adverse event	81 (14)	11.6	35 (11)	5.0	0.02
Skin reactions	12 (5)	1.6	6 (4)	0.9	0.79
Dizziness	4 (2)	0.5	2 (2)	0.3	1.00
Headache	26 (6)	3.5	6 (3)	0.9	< 0.01
General malaise	17 (6)	2.3	2 (2)	0.3	< 0.01
Fatigue	18 (5)	2.4	8 (3)	1.1	0.36
Increased hunger sensation	4 (1)	0.5	3 (1)	0.4	0.43
Cramps	1 (1)	0.1	5 (4)	0.7	0.03
Diarrhea	0 (0)	0.0	1 (1)	0.1	0.39
Dry mouth	0 (0)	0.0	1 (1)	0.1	0.39
Nausea	0 (0)	0.0	1 (1)	0.1	0.39
Lumbago	1 (1)	0.1	0 (0)	0.0	> 0.99
Palpitations	1 (1)	0.1	0 (0)	0.0	> 0.99
Hypertension	3 (2)	0.4	0 (0)	0.0	0.28
Local reactions at injection site	0 (0)	0.0	23 (11)	3.3	< 0.01
Serious adverse event	3 (2)	0.1	0 (0)	0.0	0.29

Frequency, absolute frequency of adverse events, in brackets are the unique patients; rate, number of adverse events/per person-year; *P* value, comparison of absolute frequency of adverse events with IVIg and fSCIg; fSCIg, human immune globulin 10% with recombinant human hyaluronidase; IVIg, intravenous immunoglobulins

During the study, three serious adverse events (coronary artery disease, ischemic stroke and diabetes mellitus) occurred in two patients (Table 2). Thrombosis is a rare adverse event of immunoglobulin treatment. However, all serious adverse events were considered unrelated to fSCIg treatment. The first patient reported angina pectoris at visit 4, during fSCIg treatment, but, in retrospect, this complaint had already been present 3 months before the start of the study (during treatment with IVIg), and had not been reported at visits 1–3. After cardiological evaluation, coronary artery disease was diagnosed. The cardiovascular risk profile of this patient consisted of hypertension, hypercholesterolemia, recurrent transient ischemic attacks (TIAs) treated with carotid endarterectomy and smoking. Between visits 6 and 7 (during IVIg treatment), the same patient had been admitted to hospital because of new-onset diabetes mellitus. The second patient reported headache and visual complaints, i.e., spots in the left visual field, after only one low dose of fSCIg (10 g) combined with a regular high dose of IVIg (40 g). MRI cerebrum showed a small occipital lobe infarction. After extensive workup performed by a neurovascular specialist, a combination of cardiovascular risk factors (hypercholesterolemia, hypertension and smoking) was deemed to be the most likely cause. During follow-up, this patient made a full recovery. Recovery of the visual field was confirmed by a normal perimetry examination performed by an ophthalmologist.

### Muscle strength and disability

Overall, there were no significant differences between fSCIg and IVIg expressed in vigorimetry, 9-HPT, MRC sum score or HHD total score (Tables 3, 4). Interestingly, there was a strong improvement over time in the 10-meter walk test (both in time taken and number of steps, *p *values < 0.001). This observation may suggest a learning effect over time. Despite the adjustment for time, this learning effect might obscure accurate estimation of the difference between fSCIg and IVIg in the 10-meter walk test. The SES increased by 0.6 points (95% CI 0.1–1.2, *p* = 0.021) when switching to fSCIg. The deterioration in SES is temporary and improvable as it is most likely caused by a decline in muscle strength of one patient at visit 5, with a normalisation of the score when the dose of fSCIg was increased. Excluding this patient results in an increase in SES of 0.4 points (95% CI − 0.1 to 0.8, *p *value = 0.097).

**Table 3:** Longitudinal outcome measures

Endpoint	Intercept	Treatment (IVIg vs. fSCIg)			Time (Months)		
		Coefficient	95% CI	*p* value	Coefficient	95% CI	*p* value
Vigorimetry, kPa	131.2	− 2.9	− 12.2 to 6.4	0.54	0.5	− 0.7 to 1.6	0.41
MRC sum (0–180)	163.4	− 0.8	− 2.2 to 0.7	0.30	0.2	0.0 to 0.3	0.051
SES	11.4	0.6	0.1 to 1.2	0.021	0.0	0.0 to 0.1	0.36
10-meter walk, steps	14.0	0.3	0.1 to 0.6	0.020	− 0.1	− 0.1 to 0.0	< 0.001
10-meter walk, s	8.4	0.3	0.0 to 0.5	0.040	− 0.1	− 0.1 to − 0.1	< 0.001
9-hole peg, s	32.3	2.5	0.0 to 5.1	0.051	− 0.2	− 0.6 to 0.1	0.12
Treatment satisfaction	7.9	− 0.5	− 1.0 to 0.0	0.067	0.0	− 0.1 to 0.1	0.82

Results are given per mixed model with a fixed effect for treatment and a random intercept per individual (*n* = 17), adjusted for time to account for potential disease progression during study follow-up. The treatment estimate is the mean difference between treatment arms

fSCIg, human immune globulin 10% with recombinant human hyaluronidase; IVIg, intravenous immunoglobulins; kPa, kilopascals; MRC, medical research council scale; SES, self-evaluation scale

**Table 4:** Mean difference in hand-held dynamometry

Endpoint	Mean difference (post–pre)	95% CI	*p * value
Dynamometry, Newton			
Total	− 21.3	− 75.8 to 33.3	0.42
Shoulder abduction	− 11.4	− 33.0 to 10.2	0.28
Biceps flexion	− 1.1	− 11.5 to 9.2	0.82
Wrist extension	1.5	− 7.2 to 10.2	0.72
Finger extension	2.5	− 4.5 to 9.5	0.46
Finger spreading	2.5	0.0 to 5.0	0.049
Thumb abduction	− 1.8	− 4.5 to 0.9	0.18
Hip flexion	− 4.8	− 16.3 to 6.7	0.38
Ankle flexion	− 7.5	− 24.8 to 9.8	0.37
Toe extension	− 1.2	− 9.9 to 7.6	0.78

Two patients were excluded due to missing data of fSCIg. The mean difference was calculated as the difference between first evaluation under fSCIg (visit 4) and baseline (visit 1)

95% CI , 95% confidence interval

### Treatment satisfaction and reasons for continuation

Overall, treatment satisfaction remained unchanged. The average treatment satisfaction with regard to IVIg and fSCIg was 7.9 (95% CI 7.3–8.5) and 7.5 (95% CI 6.8–8.1), respectively. Main reasons for continuation of fSCIg were independence to administer treatment (*n* = 8) and decrease in presence of adverse events [general malaise (*n* = 1), skin reaction (*n* = 1)].

## Discussion

In the present study, we showed that the safety of fSCIg, a new mode of treatment, is comparable to IVIg, with the advantages of higher doses and less frequent infusion compared to conventional SCIg. In The Netherlands, approximately 90% of the patients with MMN are treated in a home care program, in contrast to countries where IVIg treatment is only given in a hospital setting. For this reason, the satisfaction rate for IVIg treatment is high, as there is no burden of travelling to hospital. Nevertheless, fSCIg was preferred compared to IVIg treatment by almost half of the patients, and they continued with fSCIg after study closure, in particular because of independence and flexibility to administer treatment and a decrease in systemic adverse events. A significant number of patients remained on IVIg treatment, probably because the benefits of fSCIg (i.e., more independence and flexibility of administration and a decrease in systemic adverse events) did not outweigh the well-facilitated IVIg home program due to the local reactions at fSCIg injection site. Moreover, in countries which do not offer the option of IVIg treatment in home setting, fSCIg could be an even more favorable option.

Regarding safety of fSCIg, we found similar results compared to previous publications on SCIg in MMN or to fSCIg in primary immunodeficiencies, and to a recently published study that compared fSCIg with conventional SCIg in 20 patients with MMN [5–7, 9, 11, 13–16]. We reported local reactions at the injection sites in 64.7% of the patients, which is in accordance with previous studies that described local adverse reactions of fSCIg in 44–100% of the patients [5–7, 16, 17]. A systematic review and meta-analysis reported a significant reduction of 28% in the relative risk ratio of systemic adverse events of SCIg compared to IVIg; this is comparable to a significant reduction in systemic adverse events of fSCIg versus IVIg in our study [15]. Overall, similar to previous studies, muscle strength, disability and treatment satisfaction in our study remained stable, showing equal muscle strength and disability and unchanged or improved quality of life and treatment satisfaction for SCIg compared to IVIg in patients with MMN [5–7, 13, 14, 16]. Therefore, fSCIg could be a favorable alternative to IVIg treatment in MMN, as systemic adverse events may decrease, muscle strength, disability and treatment satisfaction remains stable, and there is the advantage of independence and flexibility of administration. Moreover, professional supervision of administration is not necessary for fSCIg treatment and could, therefore, reduce medical costs [17–19].

An advantage of treatment with fSCIg is the reduced number of infusions compared to SCIg. This is relevant since we have previously found that patients in The Netherlands preferred IVIg in home setting to SCIg because of the high number of infusions of SCIg (unpublished data). This is in accordance with the results of a randomized single blinded cross-over trial and follow-up study by Harbo et al., investigating SCIg versus IVIg [6, 20]. In this study, 4/9 patients preferred IVIg to SCIg especially because of the significantly lower number of infusions. Additionally, fSCIg allows self-administration of loading doses if necessary, as opposed to SCIg treatment, which requires IVIg loading doses and hence loss of independence and flexibility of administration [7, 21].

No clinical outcomes were associated with an increased risk of discontinuing fSCIg. Remarkably, the only prognostic factor for continuation of fSCIg was a higher (≥ 8) satisfaction in the IVIg phase of the study. These findings may be explained by the expectation level of patients regarding treatment with fSCIg. Patients who were less satisfied with IVIg treatment may have had higher expectations of fSCIg treatment, but as muscle strength, disability and treatment satisfaction were comparable to IVIg, these expectations may not have been met, causing patients to discontinue fSCIg earlier. Interestingly, in this study, patients who continued with fSCIg after study closure were more satisfied compared to their previous IVIg treatment because of the independence and flexibility of administration.

Study limitations include the relatively small number of patients, a common challenge in studies on rare disorders such as MMN. We were able to contact 48 patients with MMN of whom 18 (38%) participated. Furthermore, the study design was a prospective cohort and not a randomized controlled blinded trial. However, the route of administration of fSCIg did not allow a blinded study design, and as IVIg is standard of care this would limit the possibility withholding patients from immunoglobulin treatment. Moreover, we believe this study design was adequate for our aim to explore whether fSCIg could replace IVIg in individual patients, and whether it could serve as an alternative route of administration in a relatively rare disorder.

In conclusion, our study shows that safety of fSCIg is comparable to IVIg. Overall muscle strength, disability and treatment satisfaction remained unchanged after switch to fSCIg. Therefore, fSCIg could be a favorable option in patients who prefer self-treatment and more independency, and in patients who experience systemic adverse events on IVIg or have difficult intravenous access.

## Electronic supplementary material

Below is the link to the electronic supplementary material.

Online Resource 1: Proportion of patients remaining on fSCIg treatment. (A) Kaplan-Meier curve of the proportion of patients on fSCIg treatment, the median time on fSCIg treatment was 244 days (n=17); for patients that continued with fSCIg 267 days and patients that discontinued with fSCIg 37 days. (B) For each patient, the average treatment satisfaction score on IVIg was calculated during phase 1 (visit 1-3) and assessed in a Cox proportional hazards models (HR 0.31 95% CI 0.12 – 0.83, p-value = 0.007). To visualize its effects, we created two subgroups (green line; higher satisfaction level on IVIg and red line; lower satisfaction level on IVIg) based on the median of this satisfaction level. fSCIg = Human Immune Globulin 10% with recombinant human Hyaluronidase; IVIg = intravenous immunoglobulins (PDF 238 kb)

## Data Availability

The data that support the findings of this study are openly available in clinical trials database at https://clinicaltrials.gov; reference number: NCT02885259.
