# Supplementary material for: Human immune globulin 10% with recombinant human hyaluronidase in multifocal motor neuropathy
Source: J Neurol. 2019 Jul 19;266(11):2734–42. doi: 10.1007/s00415-019-09475-x (PMC6803588; doi:10.1007/s00415-019-09475-x)
Supplement: Supplementary file 1 — Online Resource 1: Proportion of patients remaining on fSCIg treatment. (A) Kaplan-Meier curve of the proportion of patients on fSCIg treatment, the median time on fSCIg treatment was 244 days (n=17); for patients that continued with fSCIg 267 days and patients that discontinued with fSCIg 37 days. (B) For each patient, the average treatment satisfaction score on IVIg was calculated during phase 1 (visit 1-3) and assessed in a Cox proportional hazards models (HR 0.31 95% CI 0.12 – 0.83, p-value = 0.007). To visualize its effects, we created two subgroups (green line; higher satisfaction level on IVIg and red line; lower satisfaction level on IVIg) based on the median of this satisfaction level. fSCIg = Human Immune Globulin 10% with recombinant human Hyaluronidase; IVIg = intravenous immunoglobulins (PDF 238 kb) [file 415_2019_9475_MOESM1_ESM.pdf]

**Title:** Human Immune Globuline 10% with Recombinant Human Hyaluronidase in Multifocal Motor Neuropathy

**Authors:** Ingrid J.T. Herraets, MD; Jaap N.E. Bakers; Ruben P.A. van Eijk, MD; H. Stephan Goedee, MD, PhD; W. Ludo van der Pol, MD, PhD; Leonard H. van den Berg, MD, PhD

**Journal name:** Journal of Neurology

**Corresponding Author:** Leonard H. van den Berg, UMC Utrecht, Department of Neurology, l.h.vandenberg@umcutrecht.nl

**Online Resource 1** Proportion of patients remaining on fSCIg treatment

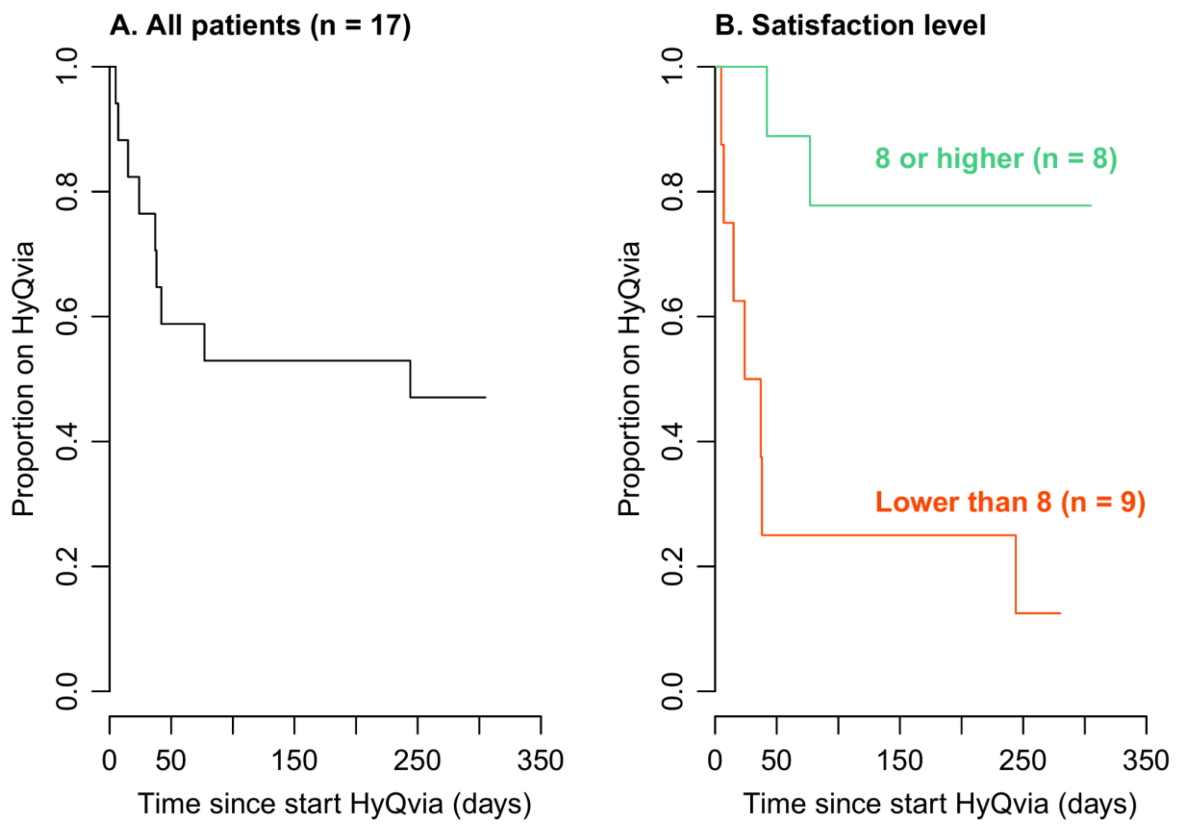


(A) Kaplan-Meier curve of the proportion of patients on fSCIg treatment, the median time on fSCIg treatment was 244 days (n=17); for patients that continued with fSCIg 267 days and patients that discontinued with fSCIg 37 days. (B) For each patient, the average treatment satisfaction score on IVIg was calculated during phase 1 (visit 1-3) and assessed in a Cox proportional hazards models (HR 0.31 95% CI 0.12 – 0.83, *p*-value = 0.007). To visualize its effects, we created two subgroups (green line; higher satisfaction level on IVIg and red line; lower satisfaction level on IVIg) based on the median of this satisfaction level.
fSCIg = Human Immune Globulin 10% with recombinant human Hyaluronidase; IVIg = intravenous immunoglobulins.
